# Anti-inflammatory effects of triptolide improve left ventricular function in a rat model of diabetic cardiomyopathy

**DOI:** 10.1186/1475-2840-12-50

**Published:** 2013-03-25

**Authors:** He-Ling Wen, Zhong-Shu Liang, Rui Zhang, Kan Yang

**Affiliations:** 1 Department of Cardiology, The Third Xiangya Hospital of Central South University, No. 138 Tongzipo Road, Changsha, Hunan, 410013, P.R.China; 2 Department of Cardiology, Sichuan Academy of Medical Sciences & Sichuan Provincial People’s Hospital, No.32 West Second Section First Ring Road, Chengdu, Sichuan, 610072, P.R.China; 3 Department of Cardiovascular and Thoracic Surgery, The Seventh People’s Hospital of Chengdu, No. 1 Twelfth Middle Street, Chengdu, Sichuan, 610041, P.R.China

**Keywords:** Triptolide, Diabetic cardiomyopathy, NF-κB, Inflammation

## Abstract

**Aims:**

Given the importance of inflammation in the onset and progression of diabetic cardiomyopathy, we investigated the potential protective effects of triptolide, an anti-inflammatory agent, in streptozotocin-induced diabetic rat model and in H9c2 rat cardiac cells exposed to high glucose.

**Methods and results:**

Diabetic rats were treated with triptolide (100, 200, or 400 μg/kg/day respectively) for 6 weeks. At the end of this study, after cardiac function measurements were performed, rats were sacrificed and their hearts were harvested for further histologic and molecular biologic analysis. Enhanced activity and expression of nuclear factor-kappaB (NF-κB) p65 in diabetic hearts were associated with increased inflammatory response, as demonstrated by increased pro-inflammatory cytokines, cell adhesion molecules and invading inflammatory cells, as well as increased fibrosis, in line with impaired left ventricular function. Triptolide attenuated these morpho-functional alterations. Furthermore, triptolide (20 ng/ml) also attenuated high glucose-induced inflammation in H9c2 rat cardiac cells.

**Conclusion:**

Our data demonstrate that anti-inflammatory effects of triptolide involving the NF-κB signaling pathway can improve left ventricular function under diabetic conditions, suggesting triptolide treatment might be beneficial in diabetic cardiomyopathy.

## Introduction

Diabetic cardiomyopathy, one of the leading cardiovascular complications in diabetic patients, has gained much interest due to its subsequent heart failure and eventually increased mortality. Over the last decades, accumulating evidence from both clinical data and animal models shows that diverse mechanisms are involved in the development of diabetic cardiomyopathy, including microangiopathy, alterations in substrate metabolism, oxidative damage, cardiac inflammation and fibrosis [1-4]. Among these abnormalities, the unresolved inflammatory response plays a key role in the onset and progression of diabetic cardiomyopathy [5,6]. Chronic inflammation could directly and indirectly cause cardiac tissue injury such as myocardial fibrosis, necrosis and apoptosis, which inevitably leads to left ventricular (LV) diastolic and then systolic dysfunction. The recognition of the inflammatory basis for diabetic cardiomyopathy may greatly contribute to the management of this disease.

*Tripterygium wilfordii Hook F* (TwHF), due to its well-established cost-effect ratio, has been used in traditional Chinese medicine to treat autoimmune and inflammatory diseases for centuries [7,8]. Triptolide (TP), a purified component of TwHF, accounts for its major bioactive effect. Randomized controlled clinical trails in the United States have shown the safety and efficacy of triptolide in treating patients with rheumatoid arthritis [9-11]. Triptolide has also been used to treat other immune-mediated inflammatory diseases, such as complex nephritis and systemic lupus erythematosus. In addition, it has been demonstrated triptolide can effectively prolong the cardiac allograft survival [12,13], so the clinical applications of triptolide have been extended to organ transplantation.

A recent study has shown that triptolide, through suppressing renal inflammation and oxidative stress, prevents diabetic nephropathy progression [14]. Therefore, on the basis of the above considerations, we hypothesized that triptolide may exert protective effects in a rat model of diabetic cardiomyopathy and in cultured cardiomyocytes exposed to high glucose (HG).

## Methods

### Animal model and treatment

Diabetes mellitus (DM) was induced in 8-week-old male Sprague–Dawley (SD) rats (Central South University Animal Centre, China) by a single intraperitoneal injection of streptozocin (STZ, 70 mg/kg, dissolved in 0.1 M sodium citrate buffer, pH 4.5; Sigma, USA) after starvation overnight. Three days and one week after the injection, random blood glucose level was measured using Onetouch SureStep glucometer (LifeScan, USA) by tail vein blood sampling. Only rats with blood glucose level > 16.7 mmol/l in both tests were selected in this study. These 1-week diabetic rats were randomly divided into four groups (n = 10 each): three diabetic groups treated with triptolide (100, 200, or 400 μg/kg/day respectively) and one diabetic group treated with vehicle. In order to verify the potential side effects of triptolide, one sex- and age-matched non-diabetic group (SD rats injected with sodium citrate buffer only, n = 10) was treated with high-dose triptolide (400 μg/kg/day). Triptolide (Chinese National Institute for the Control of Pharmaceutical and Biological Products, China), dissolved in dimethylsulfoxide (DMSO; Sigma, USA), was given by daily gastric gavage for 6 weeks. Another sex- and age-matched non-diabetic group (n = 10) was referred to as the control group. Rats were kept in the laminar flow cages on a 12 h/12 h dark/light cycle with free access to standard chow and tap water. At the end of this study, after cardiac function measurements were obtained, rats were sacrificed and their hearts were harvested for further histologic and molecular biologic analysis. In addition, blood from the aorta was collected for the determination of liver and renal functions, as well as serum marker of cardiac damage. The investigation was approved by the Institutional Animal Care and Use Committee of Central South University.

### Cardiac function measurement

Echocardiography was performed in all rats using GE Vivid 7 ultrasound system with a 10-MHz transducer (General Electric, USA). During the procedure, rats were anesthetized with intraperitoneal injection of pentobarbital (50 mg/kg) and placed in the supine position. LV end-diastolic dimension (LVEDD) and LV end-systolic dimension (LVESD) were measured on the parasternal LV long axis view. These chamber dimensions were indexed to body weight. LV ejection fraction (LVEF) and fractional shortening (FS) were calculated by assuming a spherical LV geometry with the algorithms of ultrasound system. The parameters above were measured at least three times and averaged. All measurements were performed by an experienced investigator who was blinded to the grouping and treatment information.

### Histopathology and immunohistochemistry

Heart samples were fixed in 4% buffered paraformaldehyde solution and embedded in paraffin. The 5 μm tissue sections were stained with Sirius red, assessing for total cardiac collagen content. For immunohistochemistry, after blocking endogenous peroxidase activity with 3% hydrogen peroxide, the sections were incubated with blocking buffer to further block unspecific sites. Staining was performed with the following primary antibodies at the given dilution overnight at 4°C: collagen I (Col I, 1: 200; Abcam, USA), collagen III (Col III, 1: 200; Abcam, USA), CD68 (1: 100; Adb Serotec, Germany) and CD3 (1: 300; Adb Serotec, Germany). After washing, the sections were incubated with biotin-labeled secondary antibody (1: 400; Vector lab, USA) for 1 hour at room temperature followed by color development with DAB kit (Vector lab, USA). Immunohistochemical staining was quantified with Image Pro Plus 6.0 software on 10 fields of the left ventricle.

### Quantitative real-time RT-PCR

Quantitative real-time RT-PCR was performed to assess transcript levels of tumor necrosis factor-α (TNF-α), interleukin-1β (IL-1β), intercellular adhesion molecule-1 (ICAM-1), vascular cell adhesion molecule-1 (VCAM-1) and nuclear factor-kappaB (NF-κB) p65. After RNA extraction, the concentration and integrity of the RNA was determined. Total RNA was then reverse-transcribed to cDNA, and the target genes were amplified using the type of CFX96 real-time PCR system (Bio-Rad, USA). The amplification conditions were set as followings: initial hold steps (50°C for 2 min, then 95°C for 10 min) and 40 cycles of a two-step PCR (95°C for 15 s and 60°C for 1 min). The fold change of the target PCR product was calculated after adjusting for β-actin using the comparative delta-delta C_t_ method. The primers used in this study were obtained from Genscript Corp (Nanjing, China), and its sequences were as follows: for TNF-α 5^′^-TGACCCCCATTACTCTGACC-3^′^ and 5^′^-GGCCACTACTTCAGCGTCTC-3^′^; for IL-1β 5^′^-CTCCATGAGCTTTGTACAAGG-3^′^ and 5^′^-TGCTGATGTACCAGTTGGGG-3^′^; for ICAM-1 5^′^-GTCTCATGCCCGTGAAATTATG-3^′^ and 5^′^-CATTTTCTCCCAGGCATTCTCT-3^′^; for VCAM-1 5^′^-GGAGGTCTACTCATTCCCTGAAGA-3^′^ and 5^′^-ACCGTGCAGTTGACAGTGACA-3^′^; for NF-κB p65 5^′^-CTCCCGGGCAGGTCTCAGC-3^′^ and 5^′^-GAAACGCATGCCCCGCTGCT-3^′^; for β-actin 5^′^-CATCCTGCGTCTGGACCTGG-3^′^ and 5^′^-TAATGTCACGCACGATTTCC-3^′^.

### Western blot

Western blot was used to quantify protein levels of TNF-α, IL-1β, ICAM-1, VCAM-1 and NF-κB p65. After the measurement of protein concentration, equal amount of protein was separated by 10% SDS-PAGE and transferred to polyvinylidene fluoride membranes. The membranes were blocked with 5% nonfat milk in TBS buffer. Then different primary antibodies, including TNF-α (Bioworld, USA), IL-1β (Proteintech, USA), ICAM-1(Abcam, USA), VCAM-1 (Abcam, USA) and NF-κB p65 (Bioworld, USA), were added at a dilution of 1:1,000 overnight at 4°C. After washing, the membranes were incubated with horseradish peroxidase-conjugated secondary antibody (1: 4,000; Proteintech, USA) for 1 hour at room temperature. The blots were visualized using ECL kit (Thermo pierce, USA). β-actin was used as a loading control. The density of bands was quantified with Image Pro Plus 6.0 software.

### TransAM™ NF-κB p65 Elisa

TransAM™ NF-κB p65 Elisa kit (Active Motif, USA) was tested for the DNA-binding activity of free NF-κB p65 in nuclear extracts. Nuclear protein extraction and NF-κB p65 activation assay were performed according to the manufacturer’s instructions.

### Cell culture

H9c2 rat cardiac cell line (ATCC, USA) was cultured in DMEM in 5% CO_2_ at 37°C. The cells were divided into three groups and cultured for 48 hours: (a) D-glucose (5.5 mmol/l); (b) high D-glucose (33 mmol/l); (c) co-stimulated with high D-glucose (33 mmol/l) and triptolide (20 ng/ml). Then, the cells were harvested and processed for the molecular biologic assay. The mRNA and protein expressions of TNF-α and NF-κB p65 were analysed by quantitative real-time RT-PCR and western blot, respectively. The activity of NF-κB p65 was assessed by TransAM™ NF-κB p65 Elisa.

### Statistical analysis

SPSS software version 18.0 was used for statistical analysis. Continuous variables were presented as mean ± standard deviation (SD). Comparisons of continuous data were carried out with one-way ANOVA and post hoc analysis with Bonferroni test. When heterogeneity of variance was present, comparisons were performed with Mann–Whitney test, followed by post hoc analysis with Kruskal-Wallis test. A value of P < 0.05 was considered statistically significant.

## Results

### Characterization of animal groups

At the end of the study, the untreated and triptolide-treatment diabetic groups displayed severe hyperglycemia and higher heart weight to body weight ratio compared with non-diabetic groups. The increased ratio in diabetic groups was mainly due to the significantly smaller body weight of these animals. Triptolide treatment did not affect the above metabolic parameters in either non-diabetic or diabetic groups (Table 1).

**Table 1:** Animal characterization

	**Con**	**TP**	**DM**	**DM + TP,L**	**DM + TP,M**	**DM + TP,H**
Glucose, mmol/l	6.6 ± 1.9	5.9 ± 1.4	32.2 ± 2.5*	32.1 ± 3.5*	30.8 ± 3.0*	32.6 ± 2.6*
BW, g	464.0 ± 21.2	468.0 ± 19.6	216.3 ± 21.7*	234.8 ± 37.5*	235.7 ± 30.5*	224.3 ± 29.3*
HW, mg	1183.3 ± 14.2	1202.5 ± 12.4	758.6 ± 13.6*	762.3 ± 15.3*	771.3 ± 14.7*	734.8 ± 13.2*
HW/BW, mg/g	2.55 ± 0.32	2.56 ± 0.23	3.49 ± 0.35*	3.24 ± 0.41*	3.25 ± 0.34*	3.28 ± 0.42*

*BW* body weight, *HW* heart weight, *TP, L* low-dose triptolide (100 μg/kg/day), *TP, M* medium-dose triptolide (200 μg/kg/day), *TP, H* high-dose triptolide (400 μg/kg/day). *P < 0.05 versus Con; ^#^P < 0.05 versus DM.

### Cardiac performance

Although echocardiography did not show any differences in LV end diastolic and systolic dimensions among groups, the LV dimension indices were significantly higher in diabetic groups, indicating LV dilatation in diabetic rats. Importantly, triptolide treatment significantly improved LV dysfunction in diabetic rats, as shown by increased LVEF. And FS in triptolide-treatment diabetic groups also showed the upward trend compared with the untreated diabetic group, but the difference did not reach statistical significance (Table 2).

**Table 2:** Echocardiographic parameters

	**Con**	**TP**	**DM**	**DM + TP,L**	**DM + TP,M**	**DM + TP,H**
LVEDD, mm	6.5 ± 0.5	6.6 ± 0.5	5.7 ± 0.8	5.9 ± 0.4	5.5 ± 0.7	5.3 ± 1.1
LVEDD index, um/g	14.3 ± 1.2	14.4 ± 1.1	23.9 ± 3.4*	22.0 ± 2.9*	22.2 ± 2.1*	21.1 ± 4.6*
LVESD, mm	3.8 ± 0.3	3.8 ± 0.4	3.8 ± 0.6	3.7 ± 0.3	3.2 ± 0.3	3.2 ± 0.9
LVESD index, um/g	8.3 ± 0.7	8.2 ± 0.9	16.0 ± 2.5*	13.7 ± 2.2*	13.0 ± 1.1	12.6 ± 3.4
LVEF,%	78.2 ± 2.6	77.5 ± 3.1	67.8 ± 2.6*	73.7 ± 2.5	74.8 ± 3.8^#^	74.4 ± 3.8^#^
FS,%	43.2 ± 2.6	42.7 ± 3.3	35.0 ± 1.6*	39.5 ± 2.4	40.8 ± 3.3	40.0 ± 3.7

*LVEDD* left ventricular end-diastolic dimension, *LVESD* left ventricular end-systolic dimension, *LVEF* left ventricular ejection fraction, *FS* fractional shortening. *TP, L* low-dose triptolide (100 μg/kg/day), *TP, M* medium-dose triptolide (200 μg/kg/day); *TP, H* high-dose triptolide (400 μg/kg/day). *P < 0.05 versus Con; ^#^P < 0.05 versus DM.

### Cardiac fibrosis

**Figure 1:**
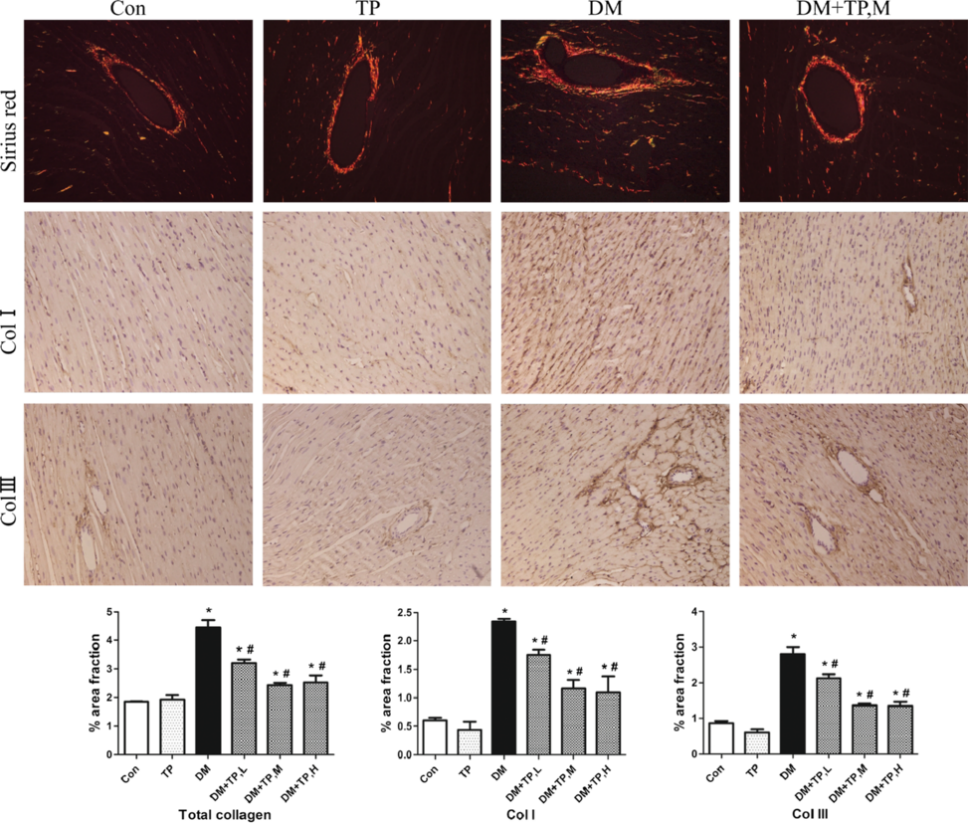
**Representative images of Sirius red staining for total collagen and immunohistochemical staining for collagen I and collagen III in cardiac tissue (200× magnification).** Bars represent quantification of expression of total collagen, collagen I and collagen III (% area fraction). TP, L, low-dose triptolide (100 μg/kg/day); TP, M, medium-dose triptolide (200 μg/kg/day); TP, H, high-dose triptolide (400 μg/kg/day). *P < 0.05 versus control; ^#^P < 0.05 versus DM, n = 10 per group.

Since collagen deposition influences the passive mechanical properties of the myocardium and then affects the cardiac performance, we observed changes of total collagen, collagen I and collagen III content in the rat hearts. Total collagen content, measured by Sirius red staining, was significantly increased in both interstitial and perivascular sites in diabetic rats compared with controls. Accordingly, collagen I and collagen III, which accounted for 90% of cardiac collagen, were increased in these rats. Triptolide treatment attenuated, but did not normalize, diabetic-induced cardiac fibrosis (Figure 1).

### Cardiac inflammation

**Figure 2:**
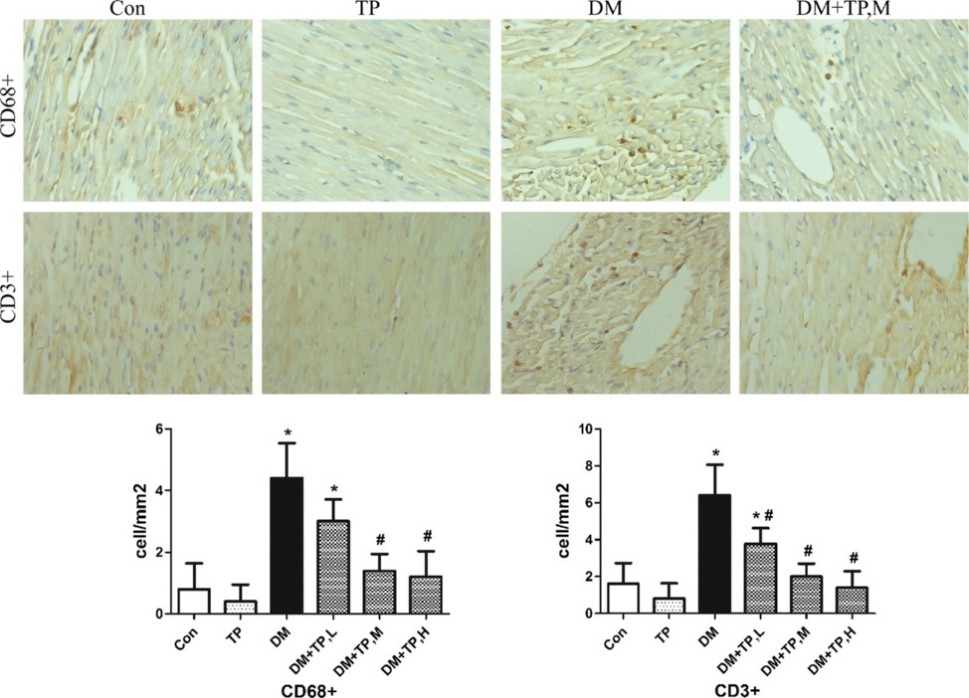
**Representative images of immunohistochemical staining for CD68+ (macrophages) and CD3+ (T lymphocytes) cells in cardiac tissue (400× magnification).** Bars represent quantification of infiltrating CD68+ and CD3+ cells (cells/mm^2^). TP, L, low-dose triptolide (100 μg/kg/day); TP, M, medium-dose triptolide (200 μg/kg/day); TP, H, high-dose triptolide (400 μg/kg/day). *P < 0.05 versus control; ^#^P < 0.05 versus DM, n = 10 per group.

**Figure 3:**
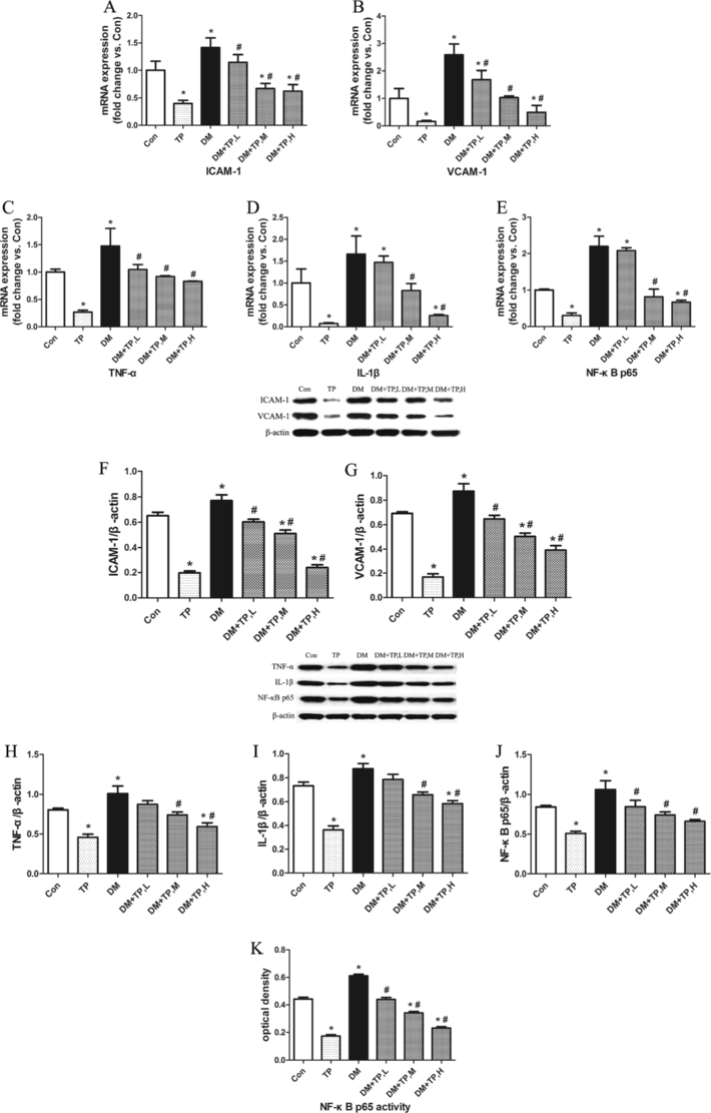
**Triptolide attenuated diabetes-induced cardiac inflammation.** (**A**-**E**) mRNA expressions of TNF-α, IL-1β, ICAM-1, VCAM-1 and NF-κB p65, as determined by quantitative real-time RT-PCR. (**F**-**J**) protein expressions of TNF-α, IL-1β, ICAM-1, VCAM-1 and NF-κB p65, as determined by western blot. (**K**) DNA-binding activity of free NF-κB p65 in nuclear extracts, as determined by TransAM™ NF-κB p65 Elisa. TP, L, low-dose triptolide (100 μg/kg/day); TP, M, medium-dose triptolide (200 μg/kg/day); TP, H, high-dose triptolide (400 μg/kg/day). *P < 0.05 versus control; ^#^P < 0.05 versus DM, n = 10 per group.

Given the importance of inflammation in the progression of diabetic cardiomyopathy, we analysed the expression of key pro-inflammatory cytokines and cell adhesion molecules, as well as the infiltration of inflammatory cells, in the cardiac tissue. The mRNA and protein levels of TNF-α and IL-1β in diabetic rats revealed a significant increase compared with controls. Enhanced expression of ICAM-1 and VCAM-1 was also observed in these rats. In agreement with increased inflammatory mediators, immunohistological staining demonstrated a higher recruitment of macrophages and T lymphocytes (CD68+ and CD3+ cells, respectively) in diabetic rat hearts. Triptolide treatment significantly attenuated the aforementioned cardiac inflammation. It should be noted that, besides its prominent anti-inflammatory effect, triptolide as an immunoregulatory agent also showed the immunosuppressive activity, thus leading to reduced expression of inflammatory mediators in non-diabetic rats (Figure 2, Figure 3A to 3D, 3F and 3I).

### Cardiac activity and expression of NF-κB p65

The transcription factor NF-κB plays a crucial role in regulating a variety of genes involved in inflammatory response. Therefore, we further examined the activity and the expression of NF-κB p65 in cardiac tissue. Consistent with the upregulated inflammatory mediators, the DNA-binding activity of free NF-κB p65 in nuclear extracts of diabetic hearts was significantly increased compared with controls. And the mRNA and protein levels of NF-κB p65 were enhanced in parallel. Triptolide treatment not only inhibited the activation of NF-κB p65, but also reduced the mRNA and protein expressions of NF-κB p65 (Figure 3E, 3J and 3K).

### Safety of triptolide treatment

To assess the safety of triptolide treatment, we tested several main indices of liver and renal functions, as well as serum marker of cardiac damage. The untreated and triptolide-treatment diabetic groups showed the elevated serum alanine aminotransferase (ALT) level compared with control group. No significant difference in aspartate aminotransferase (AST) and creatinine (Cr) levels was observed among groups. Serum creatine kinase-myoglobin (CK-MB) level in triptolide-treatment diabetic groups was not upregulted, even showed the downward trend compared with the untreated diabetic group (Table 3).

**Table 3:** Safety of triptolide treatment

	**Con**	**TP**	**DM**	**DM + TP,L**	**DM + TP,M**	**DM + TP,H**
ALT, U/l	44.7 ± 13.3	42.2 ± 12.1	72.4 ± 13.5*	76.5 ± 20.1*	84.5 ± 13.6*	89.6 ± 11.5*
AST, U/l	110.0 ± 41.0	114.0 ± 46.1	144.0 ± 59.0	141.2 ± 43.6	167.7 ± 57.3	167.4 ± 60.2
Cr, μmol/l	53.3 ± 13.8	51.5 ± 11.7	56.4 ± 15.4	41.8 ± 13.1	42.6 ± 8.2	40.2 ± 7.4
CK-MB, U/l	593.2 ± 158.1	538.2 ± 234.7	714.7 ± 132.3	322.0 ± 53.3*^#^	450.2 ± 90.8^#^	517.4 ± 79.7

*TP, L* low-dose triptolide (100 μg/kg/day), *TP, M* medium-dose triptolide (200 μg/kg/day), *TP, H* high-dose triptolide (400 μg/kg/day). *P < 0.05 versus Con; ^#^P < 0.05 versus DM.

### HG-induced inflammation in H9c2 cells

**Figure 4:**
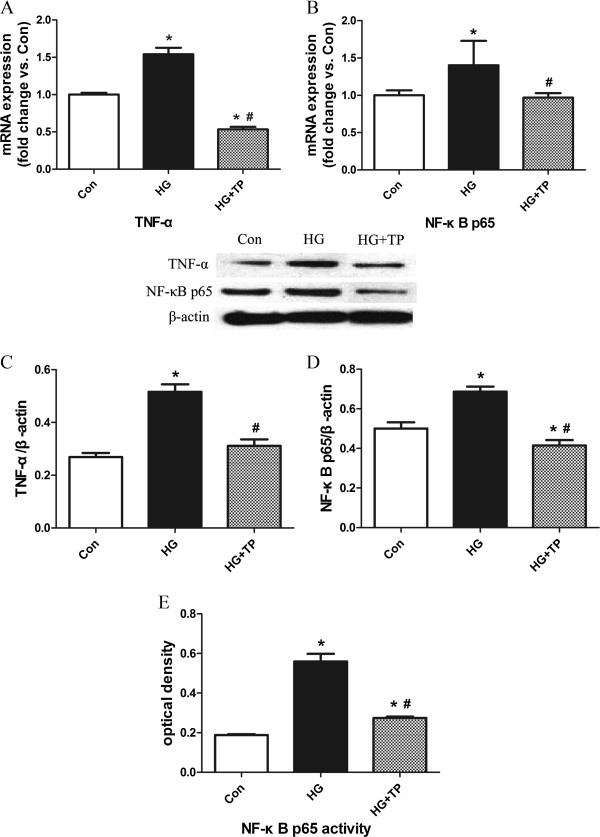
**Triptolide attenuated HG-induced inflammation in H9c2 cells.** (**A**, **B**) mRNA expressions of TNF-α and NF-κB p65, as determined by quantitative real-time RT-PCR. (**C**, **D**) protein expressions of TNF-α and NF-κB p65, as determined by western blot. (**E**) DNA-binding activity of free NF-κB p65 in nuclear extracts, as determined by TransAM™ NF-κB p65 Elisa. *P < 0.05 versus control; ^#^P < 0.05 versus HG, n = 6 per group.

H9c2 cells incubated with HG for 48 hours displayed overt inflammatory response, as shown by the enhanced activity of NF-κB p65, as well as increased mRNA and protein expressions of TNF-α and NF-κB p65. Triptolide markedly inhibited HG-induced inflammation (Figure 4).

## Discussion

To our knowledge, this study is the first to reveal that chronic treatment with triptolide attenuates cardiac inflammation and myocardial fibrosis, resulting in improved LV function in a rat model of diabetic cardiomyopathy.

### Cardiac inflammation in diabetic cardiomyopathy

Consistent with previous study, diabetic rats in our study displayed overt intramyocardial inflammation 7 weeks after STZ injection [15], as evidenced by enhanced activity and expression of NF-κB p65, thus leading to increased levels of cardiac pro-inflammatory cytokines (TNF-α, IL-1β), enhanced expressions of cell adhesion molecules (ICAM-1, VCAM-1), and activated invading immunocompetent cells (macrophages, T lymphocytes). In addition, cardiac collagen content (total collagen, collagen I and collagen III) was also significantly increased in these untreated rats. Consequently, these abnormal structural alterations in diabetic rats led to impaired cardiac performance, i.e. increased LV dimension indices, reduced LVEF and FS.

Although some researchers reported myocardial inflammation is not present in long-term experimental diabetic model [16], the notion that inflammation is an early response to diabetic insult is widely accepted. And once the advanced structural alterations are formed, the prognosis of diabetic cardiomyopathy is very poor. Therefore, the rationale supporting anti-inflammatory agents’ early usage in diabetic cardiomyopathy is 3-fold. First, inflammation characterized by elevated inflammatory cytokine production exists not only in the myocardium of animal models [15,17,18], but also in the serum of diabetic patients, even in those patients with good glycemic control [19]. Second, it has been demonstrated that inflammatory cytokines (TNF-α, IL-1β) participate in the development of LV dysfunction, and negatively correlate with LV contractility [20,21]. Inflammatory cytokines can attenuate cardiomyocyte contractility directly through the immediate reduction of systolic cytosolic calcium and indirectly through attenuation of myofilament calcium sensitivity [22], and this detrimental effect may be reversible by clearance of the cytokine exposure. Last but not the least, though the Framingham study indicated poor glycemic control was associated with an increased risk of heart failure [23], the ADVANCE (Action in Diabetes and Vascular Disease) trail and a meta-analysis unexpectedly showed intensive glycemic control did not reduce the occurrence of heart failure [24,25]. Furthermore, heart failure progression is not blocked by optimal therapy with angiotensin-converting enzyme inhibitors and beta-blockers.

As the most extensively studied cytokine, TNF-α is a noteworthy pro-inflammatory cytokine with various pathogenic effects. On one side, TNF-α is a valuable biomarker, for its correlation with enhanced brain natriuretic peptide and adverse clinical outcome in patients with heart failure [26,27]. On the other side, TNF-α is a strong mediator in the progression of heart failure. TNF-α not only exacerbates inflammatory response through acting as a signal amplifier to induce other inflammatory cytokines production, but also contributes to myocardial hypertrophy and fibrosis, leading to LV remodeling and dysfunction [28]. Thus, TNF-α may serve as a potential target in preventing LV dysfunction therapy. Although the result of anti-TNF therapy is encouraging in animal models, the anti-TNF therapy in clinical trials does not show benefits in preventing LV dysfunction [29,30]. One explanation for the negative result is that targeting a single cytokine is not an effective approach under a complex network of inflammatory signaling pathways, as other redundant cytokines may continue to propagate the inflammatory response. A more robust and comprehensive strategy would be to target a more central locus, such as NF-κB, which integrates signals from multiple inflammatory mediators [2,5].

### Therapeutic effect of triptolide

In the current study, we observed treatment with triptolide significantly attenuated cardiac inflammation and fibrosis through suppressing the activity and the expression of NF-κB in a rat model of diabetic cardiomyopathy. It is reasonable to suggest these morpho-functional changes improved, although not completely normalized, LV systolic function. In H9c2 cells exposed to HG, triptolide also markedly inhibited NF-κB activation and attenuated HG-induced inflammation. The cardioprotective effects of triptolide may be mediated, at least in part, through NF-κB signaling pathway.

As shown in the present and in previous studies, triptolide inhibited the activation and the expression of NF-κB *in vitro* and *in vivo*[31-34]. NF-κB is a pleiotropic transcription factor that controls the expression of several target genes, mainly of them involved in inflammation. Under hyperglycemic condition, the DNA-binding activity of NF-κB p65 was enhanced, and its mRNA and protein expressions were also upregulated. The NF-κB activation led to the increased expression of inflammatory mediators, including pro-inflammatory cytokines (TNF-α, IL-1β) and cell adhesion molecules (ICAM-1, VCAM-1), which contributed to the recruitment and activation of inflammatory cells (macrophages, T lymphocytes) in cardiac tissue. And some of these cytokines, such as TNF-α and IL-1β, could further stimulate NF-κB activation as a positive feedback loop. Collectively, these inflammatory mediators cooperated closely to initiate and maintain cardiac inflammation in diabetic rats. However, 6 weeks of triptolide treatment significantly inhibited the activation and the expression of NF-κB, as well as the expression of NF-κB-dependent inflammatory mediators in diabetic hearts.

In our study, increased cardiac fibrosis was noticed in STZ diabetic rats and effectively ameliorated by triptolide treatment. In addition to suppress the pro-fibrotic action of NF-κB [5], the attenuation of cardiac inflammation may be another mechanism of triptolide’s anti-fibrotic effects, since cardiac fibrosis is also partly mediated by some pro-fibrotic inflammatory cytokine, such as TNF-α and IL-1β. Furthermore, hyperglycemia induced oxidative stress is an important pathogenic factor in the development of cardiac fibrosis. There is an increasing interest in suppressing oxidative stress as an effective strategy for reducing cardiac fibrosis [35,36]. And it has been demonstrated that triptolide can significantly reduce oxidative stress [33,34]. Thus, we may speculate that the anti-fibrotic effects of triptolide are also related to its inhibitory effect on oxidative stress. However, further investigation must be performed to elucidate the exact anti-fibrotic mechanism of triptolide under diabetic conditions.

Although several researchers have reported that triptolide does not affect NF-κB DNA-binding activity [37], our findings demonstrated triptolide could inhibit hyperglycemic-induced NF-κB activation both *in vitro* and *in vivo*, confirmed by TransAM™ NF-κB p65 Elisa assay. And our results are consistent with the prevailing view regarding the effect of triptolide on NF-κB activity [8]. One plausible explanation for this discrepancy is differences in cell types, animal models, or experimental methodologies.

The dosage of triptolide was selected based on our pilot study and other studies [31,32]. It should be noted that the cardioprotective effects of triptolide under diabetic condition was not exactly dose-dependent. One possibility is that the anti-inflammatory potency of triptolide at the dosage of 200–400 μg/kg/day reaches a plateau. The safety of triptolide treatment was confirmed by serum ALT, AST, Cr and CK-MB level assay. Although the untreated and triptolide-treatment diabetic groups showed the increased ALT level compared with control group, triptolide-treatment in non-diabetic rats did not upregulate ALT level. And no significant difference in AST and Cr levels was observed among groups. Moreover, triptolide treatment did not upregulate CK-MB level. Therefore, we infer that the increased ALT level in diabetic rats was due to diabetic-induced liver damage, while triptolide itself in our study did not show liver, kidney or cardiac toxicity.

### Study limitations

Although STZ is widely used to induce diabetic animal model, a few reports have indicated that STZ itself may cause cardiac morpho-functional abnormalities [38]. And the STZ-model is associated with severe hyperglycemia and hypoinsulinemia, which is not encountered in patients with type 1 diabetes receiving exogenous insulin. However, in clinical setting diabetic patients are always accompanied with comorbidities such as hypertension and atherosclerosis, the classical STZ-model is considered as an ideal model for experimental diabetic cardiomyopathy due to its relative resistance to develop hypertension and atherosclerosis [39-41]. Therefore, this model is particular useful in exploring the effect of hyperglycemia alone on cardiac tissue, that is, the underlying mechanism of diabetic cardiomyopathy. Caution should be taken when extrapolating our findings in STZ-animal models to diabetic patients. Although triptolide treatment in non-diabetic rats did not affect cardiac performance and other metabolic parameters, the longterm consequences of its potential immunosuppressive property should be further examined with care. In addition, further study is required to evaluate the therapeutic effect of triptolide post-treatment after the establishment of diabetic cardiomyopathy.

## Conclusion

In conclusion, triptolide treatment significantly attenuates cardiac inflammation and fibrosis through suppressing the activity and the expression of NF-κB, resulting in improved LV function in experimental diabetic cardiomyopathy. Our data demonstrate that triptolide might be potentially used in conjunction with current glycemic and heart failure therapies.

## Abbreviations

DM: Diabetes mellitus; FS: Fractional shortening; HG: High glucose; ICAM-1: Intercellular adhesion molecule-1; IL-1β: Interleukin-1β; LV: Left ventricular; LVEDD: Left ventricular end-diastolic dimension; LVEF: Left ventricular ejection fraction; LVESD: Left ventricular end-systolic dimension; NF-κB: Nuclear factor-kappaB; STZ: Streptozocin; TNF-α: Tumor necrosis factor-α; TP: Triptolide; VCAM-1: Vascular cell adhesion molecule-1

## Competing interests

The authors declare that they have no competing interests.

## Authors’ contributions

H-LW participated in design of the study, carried out animal drug administration, cell culture, immunohistochemistry, RT-PCR, western blot and Elisa analysis, and drafted the manuscript. Z-SL performed cardiac function measurement, analysis and interpretation of the data. RZ contributed to interpret the data and proofread the manuscript. KY participated in design and supervision of the study, analysis and interpretation of the data, statistical analysis, and critical revision of the manuscript. All authors have seen and approved the final manuscript.
